# A stochastic model for error correction of kinetochore-microtubule attachments in budding yeast

**DOI:** 10.1371/journal.pone.0236293

**Published:** 2020-08-06

**Authors:** Anand Banerjee, Neil Adames, Jean Peccoud, John J. Tyson

**Affiliations:** 1 Department of Biological Sciences, Virginia Polytechnic Institute & State University, Blacksburg, VA, United States of America; 2 Division of Systems Biology, Academy of Integrated Science, Virginia Polytechnic Institute & State University, Blacksburg, VA, United States of America; 3 Department of Chemical and Biological Engineering, Colorado State University, Fort Collins, CO, United States of America; 4 GenoFAB Inc., Fort Collins, CO, United States of America; King’s College London, UNITED KINGDOM

## Abstract

To divide replicated chromosomes equally between daughter cells, kinetochores must attach to microtubules emanating from opposite poles of the mitotic spindle (biorientation). An error correction mechanism facilitates this process by destabilizing erroneous kinetochore-microtubule attachments. Here we present a stochastic model of kinetochore-microtubule attachments, via an essential protein Ndc80 in budding yeast, *Saccharomyces cerevisiae*. Using the model, we calculate the stochastic dynamics of a pair of sister kinetochores as they transition among different attachment states. First of all, we determine the kinase-to-phosphatase balance point that maximizes the probability of biorientation, while starting from an erroneous attachment state. We find that the balance point is sensitive to the rates of microtubule-Ndc80 dissociation and derive an approximate analytical formula that defines the balance point. Secondly, we determine the probability of transition from low-tension amphitelic to monotelic attachment and find that, despite this probability being approximately 33%, biorientation can be achieved with high probability. Thirdly, we calculate the contribution of the geometrical orientation of sister kinetochores to the probability of biorientation and show that, in the absence of geometrical orientation, the biorientation error rate is much larger than that observed in experiments. Finally, we study the coupling of the error correction mechanism to the spindle assembly checkpoint by calculating the average binding of checkpoint-related proteins to the kinetochore during the error correction process.

## Introduction

**Fig 1:**
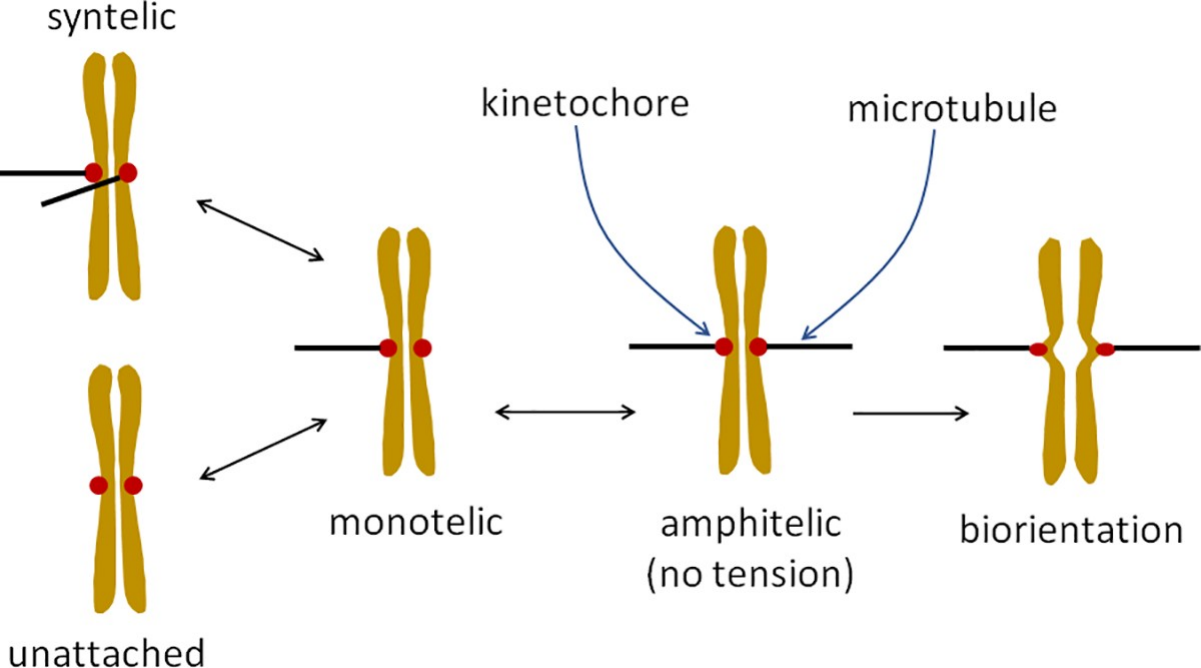
Model for transitions of sister chromatids between different KT-MT attachment states. Double-headed arrows denote reversible transitions, whereas the single-headed arrow denotes an irreversible transition from the amphitelic state (centromere not under tension) to the biorientation state (centromere under tension).

Equal partitioning of replicated chromosomes is crucial for maintaining genetic integrity from one generation to the next. A key step in this process is the attachment of the two kinetochores (KTs) on sister chromatids to microtubules (MTs) emanating from opposite poles of the mitotic spindle. The attachment process is stochastic and error prone, resulting in erroneous attachments like syntely (where both KTs are attached to the same spindle pole) and merotely (where one KT is attached to both spindle poles) (see Fig 1). Such errors must be corrected before the onset of anaphase [1–3]. The correction of erroneous KT-MT attachments in budding yeast is crucially dependent on the kinase Ipl1 (Aurora B in mammalian cells) [3–5].

The Ndc80 complex at the KT is a primary site for KT-MT attachments [6,7]. Phosphorylation of Ndc80 by Ipl1 weakens its interaction with MTs [7–9], and conversely, its dephosphorylation increases its affinity for MTs and stabilizes KT-MT attachments [10,11]. In tensionless KTs (which is the case in syntelic attachments), Ipl1 phosphorylates Ndc80, resulting in dissociation of Ndc80-MT interactions. This provides the unattached KT an opportunity to attach to a MT from the correct spindle pole [1,3,4]. Together, these observations suggest that a balance between kinase and phosphatase activities is required to break erroneous attachments and then establish correct, stable attachments between KTs and MTs [12–14]. Experiments show that a PP1 phosphatase (Glc7 is the PP1 catalytic subunit in budding yeast) opposes the kinase activity of Ipl1 [15–17], but whether it dephosphorylates Ndc80 or not and its importance in biorientation of chromosomes remain unclear.

The process of error correction is also coupled to a surveillance mechanism called the spindle assembly checkpoint (SAC). Unattached KTs, generated during error correction, initiate SAC signaling [18,19]. Briefly, unattached KTs recruit a kinase, Mps1, to phosphorylate Spc105 (Knl1 in mammalian cells) at phosphodomains called MELTs (Met-Glu-Leu-Thr sequences). Phosphorylated MELTs then bind to cytoplasmic SAC proteins to turn on the SAC signal [20]. The SAC monitors KT-MT attachments and delays the onset of anaphase until all KTs are properly attached [18,21]. Almost all the proteins involved in error correction and the SAC are conserved in yeast and humans. However, yeast KTs are smaller than mammalian KTs and attach to only one MT [22].

Although characterization of the complex network of proteins governing the error correction mechanism and the spindle assembly checkpoint has progressed greatly in recent years, dedicated models to describe the dynamics of the underlying molecular network are still lacking. Existing models on error correction are coarse-grained [23,24] and do not account for how SAC signaling is coupled to the error correction process. As a first step towards filling this gap, in this paper we present (in the context of budding yeast cells) a stochastic model to quantify the dynamics of KT-MT attachment via the protein Ndc80. Using the model, we determine (1) what ratio of kinase-to-phosphatase activities leads to maximum biorientation probability, (2) with what probability low-tension amphitelic attachments transition to the high-tension amphitelic state, (3) what are the relative contributions of Ipl1-dependent destabilization of KT-MT attachments and the geometrical orientation of KTs towards reaching biorientation, and (4) how the error correction process is coupled to the binding of essential SAC proteins to the KT.

## Model

The scheme in Fig 1 shows possible transitions between different attachment states for a pair of budding yeast KTs at the centromere of a replicated chromosome. Initial attachment of a KT to a MT occurs with the lateral surface of the MT–known as lateral attachment. The lateral attachment is then converted to an end-on attachment by sliding of the KT along the MT [2,25]. To simplify this process, we assume that the first interaction between a KT and a MT is an end-on attachment. In our model, the amphitelic state has the KTs attached to MTs from opposite poles of the mitotic spindle but the centromere is not yet under tension. The amphitelic state can transition reversibly to the monotelic state (only one KT attached to the spindle) or irreversibly to the biorientation state in which the KTs are attached to opposite poles and there is tension between the KTs. Tension is generated by the opposing forces exerted by depolymerizing MTs bound to KTs. Tension stretches the centromeric region of the chromosome and is thought to stabilize KT-MT attachments by physically separating Ipl1 from its substrates and thereby reducing its role in destabilizing KT-MT attachments [26,27]. We assume that, in the amphitelic state, Ipl1 activity remains high, which allows the possibility of detachment of a MT, and in the biorientation state it drops to zero. Hence, the probability of going from the biorientation state to the amphitelic state is zero. The criterion used to define the transition from the amphitelic to biorientation state is described later.

**Fig 2:**
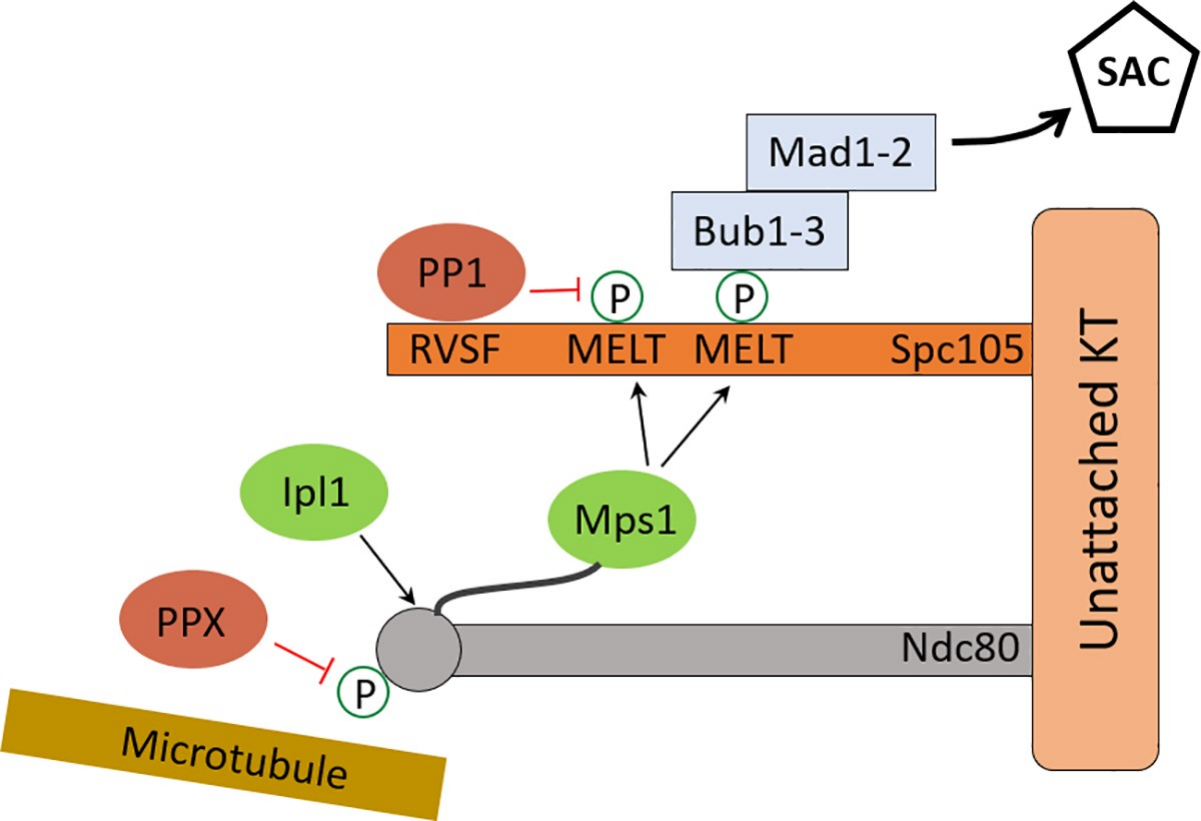
Scheme for kinase and phosphatase activities at the KT. Kinases are Ipl1 and Mps1 (shown in green), and phosphatases are PP1 and PPX (shown in red). Phosphorylation of MELT motifs by Mps1 starts the SAC signaling cascade and its dephosphorylation by RVSF-bound PP1 promotes silencing of the SAC signal. Phosphorylation of Ndc80 by Ipl1 weakens KT-MT attachment, and its dephosphorylation by PPX promotes KT-MT attachment.

Fig 2 shows the actions of kinases and phosphatases at the KT to control error correction and SAC activity, as described above. The main kinases are Ipl1 and Mps1, and the phosphatases are PP1 and PPX (an unknown phosphatase). Ipl1 phosphorylates Ndc80 [8]. At this point in time, it is not clear which phosphatase dephosphorylates Ndc80; hence, ‘PPX’ in Fig 2. Possible candidates for PPX are the free PP1 and PP2A phosphatases in the nucleus. Ndc80-bound Mps1 phosphorylates MELT repeats on Spc105 to activate SAC signaling [28], and PP1 bound to the RVSF motif dephosphorylates MELT repeats [28].

**Fig 3:**
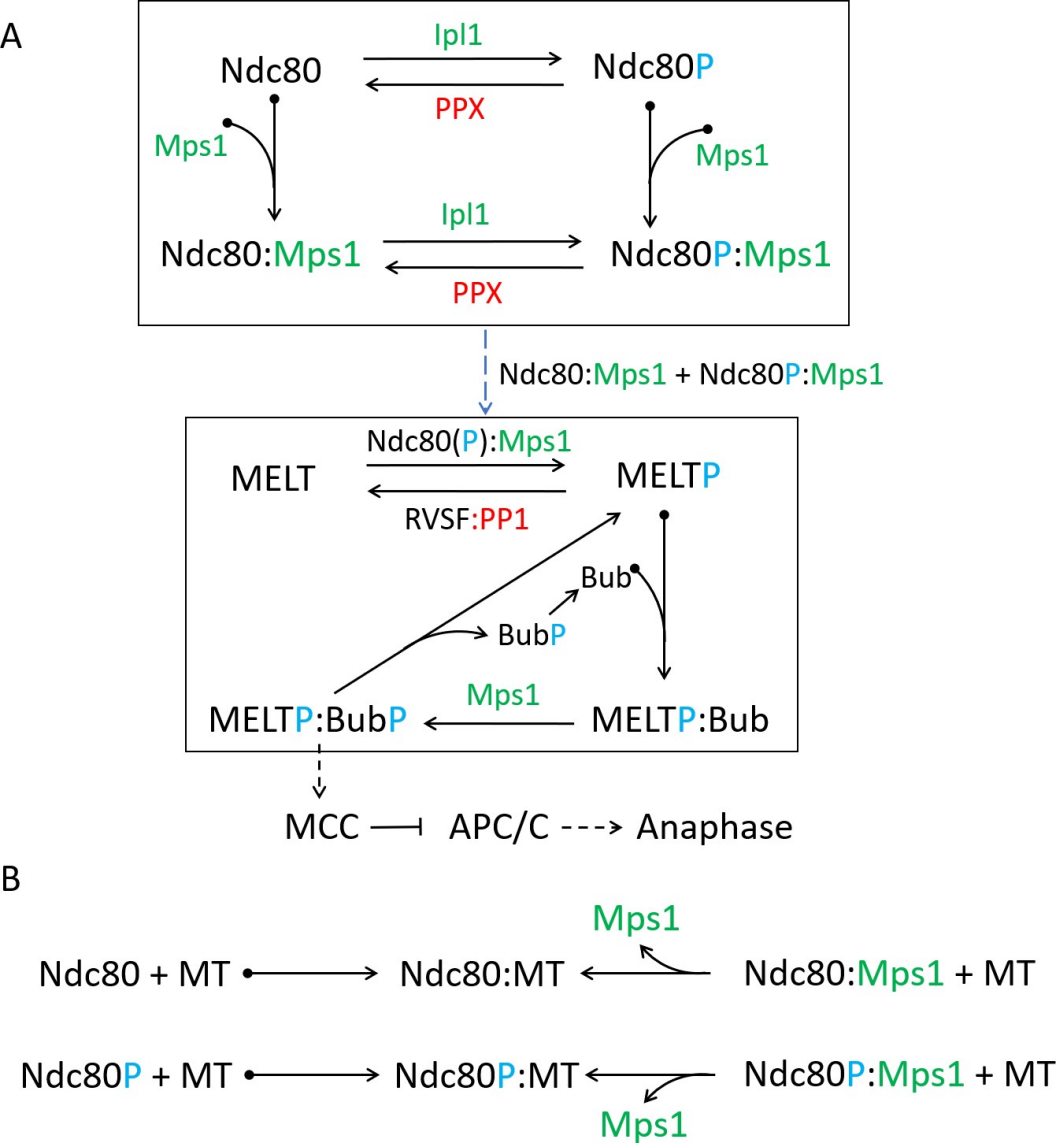
Molecular interactions at the KT. **(A)** Model of kinase (green) and phosphatase (red) activities at the KT, derived from the scheme in Fig 2. The full scheme can be divided into two modules (shown within boxes), namely, Ndc80 and MELT modules. See text for a detailed description of each module. ‘P’ (blue color) is used to depict the phosphorylation status of different motifs. The dashed arrow between the boxes indicates that Mps1 bound to either Ndc80 or Ndc80P phosphorylates MELT repeats. **(B)** Scheme showing binding of Ndc80 to MT. The reactions on the first line show that Ndc80 (unphosphorylated) binds reversibly to a MT, stabilizing the attachment, and that Ndc80:Mps1 can also bind to a MT, which displaces Mps1 in an irreversible reaction. (MTs and Mps1 compete for the same binding site on Ndc80; therefore, upon MT binding, Mps1 is displaced from Ndc80.) We assume that Ndc80P (and Ndc80P:Mps1) can also bind to MTs (second line of reactions), but with a much larger dissociation constant, i.e., phosphorylation of Ndc80 promotes detachment of the MT from a KT.

The scheme shown in Fig 2 can be understood as two coupled modules (the Ndc80- and MELT modules), whose interactions are shown in Fig 3A. The Ndc80 module consists of two phosphorylation states and two Mps1-binding states of each Ndc80 on Spc105. Ipl1 phosphorylates Ndc80 at multiple sites to modulate its interaction with MT [8]; to keep the model simple we assume only two phosphorylation states. Attachment of a MT to Ndc80 is diagrammed separately (Fig 3B). In budding yeast, approximately 7–8 molecules of Ndc80 bind to a MT [29]; in our model we choose this number to be five. The phosphatase PPX dephosphorylates Ndc80.

The second module contains different phosphorylation states and Bub-binding states of the six MELT motifs on Spc105 [30]. Assuming five Spc105 molecules per KT, there are 30 MELT motifs on each KT. We also assume that all the MELT motifs are identical. This module is crucial for generation of the SAC signal. Mps1 phosphorylates MELT motifs and RVSF-PP1 dephosphorylates them. Phosphorylated MELT repeats bind Bub3-Bub1, and then phosphorylation of Bub1 by Mps1 allows binding of Mad1-Mad2 [31]. The Mad1-Mad2 complex acts as a template for generation of the Mitotic Checkpoint Complex (MCC), a diffusible signal that delays onset of anaphase by inhibiting the Anaphase Promoting Complex/Cyclosome (APC/C; a ubiquitin ligase) [32]. We simplify this signaling cascade by lumping the SAC proteins into a single species called Bub, and assume that the state MELTP:BubP is capable of generating the MCC. The SAC signal is turned off by the dissociation of BubP from MELTP, and after dissociation BubP is dephosphorylated to Bub by some unknown phosphatase.

Coupling between the modules is shown with a dashed arrow. Mps1 bound to either Ndc80 or Ndc80P phosphorylates the MELT repeats on Spc105. We assume that a single Mps1 molecule bound to Ndc80 can phosphorylate all 30 available MELT repeats.

Our model for attachment of Ndc80 to a MT is shown in Fig 3B. KT-MT attachment is a complex process involving both the Ndc80 complex and the Dam1 complex [25]. We focus only on the Ndc80 complex and assume that both phosphorylated and unphosphorylated forms of Ndc80 can bind to a MT, and that the dissociation rate of Ndc80P:MT is much larger than that of Ndc80:MT, consistent with the observation that the affinity of Ndc80 for MTs decreases with the number of phosphorylations [9]. It is also known that MTs and Mps1 compete for the same binding site on Ndc80 [33,34]. In our model, Mps1 is displaced from Ndc80 upon MT binding.

### Kinetochore-microtubule attachment dynamics

**Fig 4:**
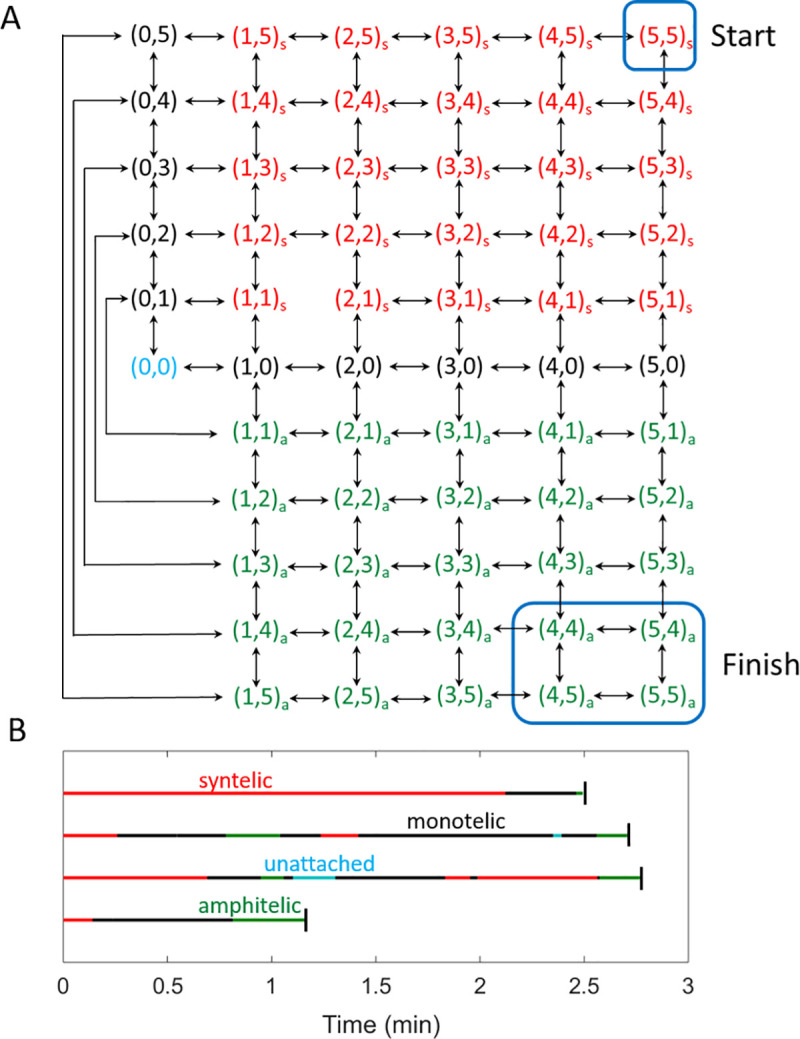
A model of MT attachment to sister KTs through Ndc80. **(A)** The vector (*m*,*n*) represents the numbers of Ndc80s bound to a MT at a pair of sister KTs. The attachment can be monotelic (black), syntelic (red with subscript s) or amphitelic (green with subscript a). The start and finish points of the chromosome-alignment process are shown with blue boxes. We assume that the process stops (KTs reach biorientation) when the KTs spend more than 1 s in the finish box without coming out. **(B)** Traces showing four time-courses of KT-MT attachment status calculated using the scheme in (A). A black vertical bar indicates the time of biorientation.

As mentioned earlier, in budding yeast, a MT attaches to a KT via Ndc80. To model the attachment dynamics we describe the attachment states of sister KTs by a two-dimensional vector (*m*,*n*), where the integers *m* and *n* correspond to the number of Ndc80s bound to a MT at each KT of a sister chromatid pair. Fig 4A shows the attachment dynamics of sister KTs. The symbols ‘s’ (red) and ‘a’ (green) correspond to syntelic and amphitelic attachments, respectively. Since KTs in budding yeast can bind only one MT at a time, merotelic attachments (a KT is attached to microtubules emanating from both spindle poles) are not possible, and not considered in our model. A double arrow between states reflects that transitions can occur in both forward and backward directions. Fig 4B shows four different realizations of the KT-MT attachment status as a function of time, calculated using the scheme in Fig 4A. All the traces start in the syntelic attachment state and end in biorientation, but, as shown later, in some cases the KTs fail to reach biorientation.

For understanding the dynamics of error correction, we choose the syntelic attachment state (5,5)_s_ as the initial state of KTs. This starting point is reasonable as syntelic attachments are frequently observed during early mitosis in budding yeast [1,2]. As mentioned earlier, sister KTs attached to MTs from opposite spindle poles (i.e., amphitelic attachments) come under tension when the MTs exert forces (due to MT depolymerization) simultaneously on both KTs of a centromere. The typical time for such an event to occur has been estimated to be ~1 second [23]. Thus, we assume that the amphitelic attachments come under tension when *x* or more Ndc80s (i.e., Ndc80+Ndc80P ≥ *x*) are bound to MTs on each side of a centromere for more than one second. For example, for *x* = 4 case shown in Fig 4A, the irreversible amphitelic-to-biorientation transition occurs when the system spends more than one second in the states (4,4)_a_, (5,4)_a_, (4,5)_a_, (5,5)_a_, without coming out of the ‘Finish’ box.

For KTs in monotelic states (black color in Fig 4), attachment of a MT to the unattached KT can be either amphitelic or syntelic (see Fig 1). This attachment is a stochastic process that depends on factors like the rotational diffusion of sister chromatids in the monotelic state. We do not account for the detailed motions of chromosomes. Instead, we take a coarse-grained approach, introducing a parameter, *P*_syn_, to specify the ratio of transitions between monotelic → syntelic and monotelic → amphitelic states. For example, *P*_syn_ = 0.5 corresponds to the case where KTs in a monotelic state are equally likely to form syntelic or amphitelic attachments. Lower values of *P*_syn_ (< 0.5) correspond to the case where KTs in a monotelic state are biased towards forming amphitelic attachments.

## Simulation

We prepared the model in an Excel file that contains the list of species, initial conditions, reactions corresponding to Ndc80 and MELT modules and KT-MT attachment, reaction-propensities, parameter values, and constraints. The list of species, initial conditions, and parameter values are also provided in S1 Table and S2 Table in S2 File.

We wrote a MATLAB code that takes the Excel file as input and outputs another MATLAB file containing the stochiometric matrix and the propensity vector, which were used to prepare the code for stochastic simulation of the model by Gillespie’s Stochastic Simulation Algorithm [35]. The Excel and MATLAB files are provided in SI. We stopped the simulation when one of the following two criteria was satisfied: (1) KTs reached biorientation, or (2) time in simulation reached 10 min. In budding yeast, the time lapse from prometaphase to anaphase is approximately 15 min [36]. During that time the KTs must become bioriented and the SAC signal in the cytoplasm must be turned off, which in budding yeast takes approximately 5 min [37]. Thus 10 min for reaching biorientation is a reasonable choice.

From our simulations we calculated the probability of biorientation within 10 min, the fraction of time spent by KTs in different attachment states, the average number of transitions between different states, and quantities related to the SAC signal. The method used to calculate these quantities is described in SI. Unless stated otherwise, we performed 10^4^ stochastic simulations to calculate the statistics at each chosen set of parameter values. To study the case where the formation of syntelic attachment is less probable than the formation of amphitelic attachment, the value of *P*_syn_ was chosen to be 0.1. Reducing *P*_syn_ to values smaller than 0.1 did not change the results significantly. *P*_syn_ = 0.5 was chosen to study the case where formation of syntelic and amphitelic attachments are equally probable. The activities of kinase and phosphatase are defined as (number of molecules)×(corresponding rate constant). For example, Ipl1 activity = #Ipl1× *k*_Ipl1_. Since the number of molecules of Ipl1 and PPX are not known, we absorb their values in the corresponding rate constants and set #Ipl1 = #PPX = 1.

## Results

### Calculation of biorientation probability in a simplified model

The dynamics of KT-MT attachment depends on the phosphorylation state of each Ndc80 and the corresponding dissociation rates. Exact values of these dissociation rates are not known. For this reason, we first consider a simplified model in which Ndc80-MT dissociation is described by a single effective parameter, *k*_d,eff_. Using this simplified model, we calculate the value of *k*_d,eff_ at which the probability of biorientation is maximum. We also analyze the effect of the criterion for biorientation by varying *x*, the minimum number of Ndc80-MT attachments needed for biorientation (*x* = 4 in Fig 4A), i.e., by changing the size of the finish box (2×2 in Fig 4A). As shown later, the value of *k*_d,eff_ determined from this analysis is useful in choosing different dissociation rates for Ndc80:MT and Ndc80P:MT attachments and in deriving an analytical formula for the kinase-phosphatase balance point. For this calculation and the next one related to kinase-phosphatase balance point, we choose *P*_syn_ = 0.1.

**Fig 5:**
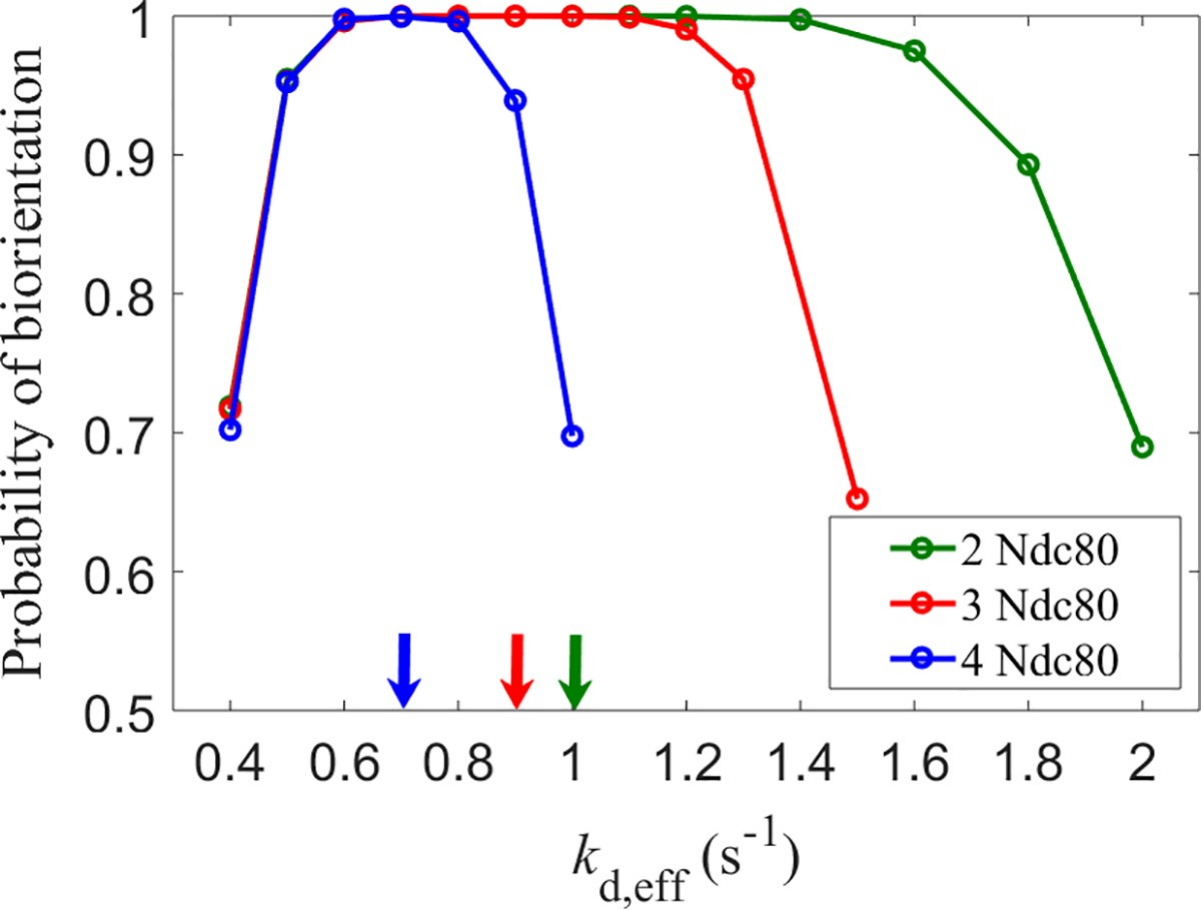
Probability of biorientation in a simplified model. Probability that sister KTs reach biorientation within 10 min, starting from the syntelic attachment state, as a function of an effective MT-Ndc80 dissociation rate (*k*_d,eff_). Different curves correspond to different numbers of Ndc80s used in the biorientation criterion. The highest value of the probability of biorientation in each case is 1 (or very close to 1). The optimal value of *k*_d,eff_, shown with an arrow, was determined using the minimum value of the mean-time for biorientation (S1 Fig in S2 File).

The probability of biorientation for different values of *x* (i.e., for different criteria for biorientation) is shown Fig 5. In each case, the biorientation probability (*P*_bio_) is approximately 1 over a range of *k*_d,eff_ values. We identify an optimal value of *k*_d,eff_ by using the minimum of the mean-biorientation-time (see S1 Fig in S2 File). As *x*, the minimum number of Ndc80-MT attachments needed for biorientation, is increased, the peak in biorientation probability shifts to the right. Interestingly, the optimal values of *k*_d,eff_ are smaller than the forward rate of Ndc80-MT binding, which is kept fixed at 2 s^−1^. To the left of the plateau, the biorientation probability drops because correction of syntelic attachments becomes inefficient. To the right of plateau, the probability drops because correct attachments are destabilized too often (see S3 Table in S2 File). Note that the optimal value of *k*_d,eff_ depends on the size of the ‘Finish’ box, which is part of the tension-dependent irreversible transition from low-tension amphitelic states to the high-tension biorientation state.

To determine the error rate, defined as 1−*P*_bio_, we performed 10^6^ simulations at the optimal value of *k*_d,eff_ (Table 1). When the minimum number of Ndc80-MT attachments required for biorientation is 3 or 4, the error rate is between 10^−6^ and 10^−4^ per chromosome, which is comparable with the mis-segregation rate per chromosome (between 10^−5^ and 10^−4^) observed in budding yeast [38]. A key factor affecting the error rate is time needed for biorientation (10 min). Increasing this time to 12 min resulted in a significant drop in the error rate in the ‘3 and 4 Ndc80’ case and no drop in the ‘5 Ndc80’ case. In all subsequent calculations the minimum number of Ndc80-MT attachments required for biorientation is chosen to be four.

**Table 1:** Optimal value of effective dissociation rate constant (*k*_d,eff_) and the error rate (1−*P*_bio_) per chromosome at that value*.

Min number of Ndc80	Optimal *k*_d,eff_ (s^−1^)	1−*P*_bio_ at 10 min	1−*P*_bio_ at 12 min
2	1	0	0
3	0.9	4×10^−6^	0
4	0.7	4×10^−4^	6×10^−5^
5	0.45	3×10^−1^	3×10^−1^

*As the minimum number of Ndc80s needed for biorientation increases, the error rate increases. Increasing the time needed for biorientation from 10 min to 12 min results in a significant drop in the error rate for the ‘4 Ndc80’ case, but no drop for the ‘5 Ndc80’ case.

### Kinase-phosphatase balance during error correction and biorientation of sister chromatids

Here we determine the ratio of kinase-to-phosphatase activities (balance point = *k*_Ilp1_/*k*_PPX_) that maximizes the biorientation probability. We focus on the dependence of the balance point on the criterion for biorientation and the dissociation rates for the Ndc80:MT and Ndc80P:MT complexes. (As mentioned earlier we consider only two phosphorylation states of Ndc80.)

The optimal value of *k*_d,eff_ determined previously (Fig 5) serves as a reference point for determining the dissociation rate constants of Ndc80:MT and Ndc80P:MT complexes (*k*_d,ndc_ and *k*_d,ndcp_, respectively). If both rates are lower or higher than the optimal value, the biorientation probability drops below the value determined using the simplified model (see S4 Table in S2 File). This suggests that *k*_d,ndc_ should be smaller and *k*_d,ndcp_ should be greater than the optimal value of *k*_d,eff_.

**Fig 6:**
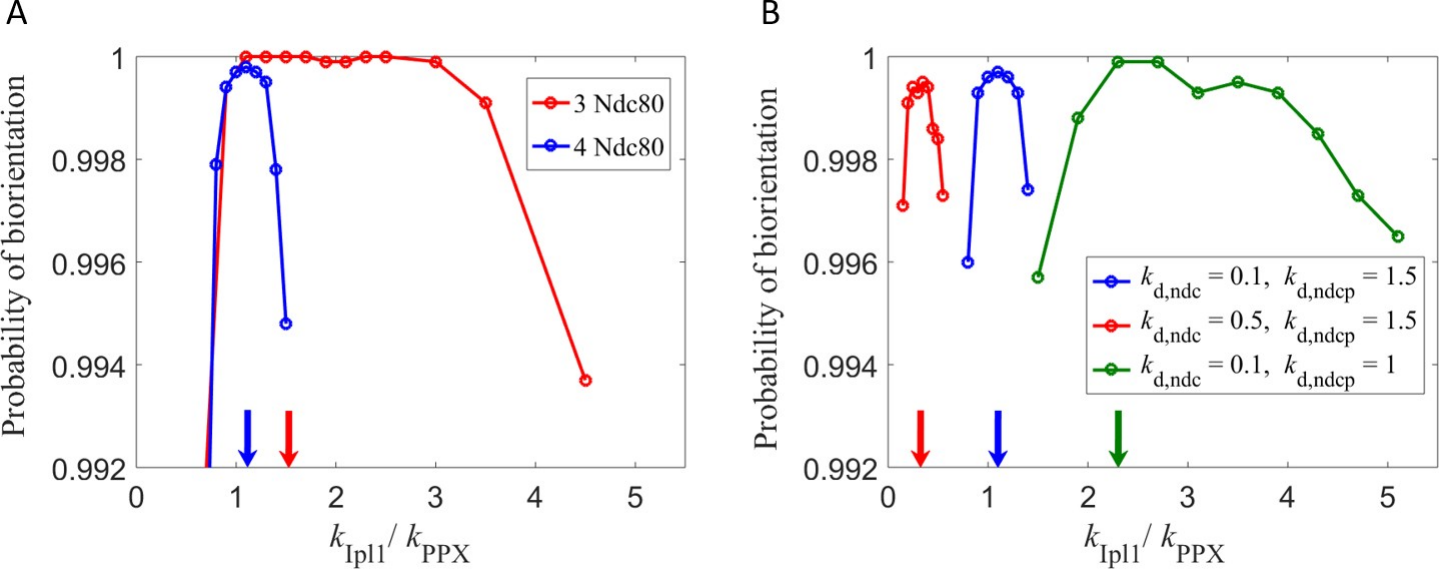
Analysis of the kinase-phosphatase balance point. Probability of sister KTs reaching biorientation within 10 min, starting from the syntelic attachment state. The location of the maximum in biorientation probability (balance point) is indicated with arrows. The value of phosphatase activity is set to *k*_PPX_ = 1 s^−1^. (A) When the minimum number of Ndc80s needed to reach biorientation is lowered, the balance point occurs at higher values of *k*_Ipl1_. For this analysis we chose Ndc80:MT and Ndc80P:MT dissociation rates as *k*_d,ndc_ = 0.1 s^−1^ and *k*_d,ndcp_ = 1.5 s^−1^ respectively. (B) Depending on the values of the MT-Ndc80 dissociation rates, the balance point occurs at values smaller or greater than one. For this analysis the minimum number of Ndc80s needed for biorientation was chosen to be four.

The probability of biorientation as a function of the ratio of kinase-to-phosphatase activities is shown in Fig 6. As in the previous analysis, below the balance point (shown with arrows) the biorientation probability drops because correction of syntelic attachments takes too long, and above the balance point the probability drops because correct attachments are destabilized too often. In budding yeast, either Ipl1 mutation or Glc7 overexpression (phosphatase-dominant conditions) increases the error rate in chromosome segregation due to excessive stabilization of KT-MT attachments [15,39]. Conversely, overexpression of Ipl1 or Glc7 mutation (kinase-dominant conditions) increases the error rate in chromosome segregation due to continuous disruption of KT-MT attachments [39,40]. The results obtained from our model are qualitatively consistent with these experimental observations.

As the criterion for biorientation is made less stringent by decreasing the number of Ndc80-MT attachments needed for biorientation, the significance of initial syntelic attachment as a barrier to reaching biorientation, increases. This barrier is more easily overcome at higher kinase activity; hence, the balance point shifts to higher values (Fig 6A). The balance point also depends on the MT-Ndc80 dissociation rates, and can occur at values smaller than or greater than one as the dissociation rates are varied (Fig 6B). A more quantitative analysis of the dependence of the balance point on these factors is done next.

### Analytical calculation of the balance point of kinase and phosphatase activities

Here we derive an approximate formula for the kinase-to-phosphatase balance point, based on the following reasoning. In the simplified model, the peak in biorientation probability occurs at an optimal value of *k*_d,eff_. Then, in general, kinase and phosphatase activities should balance to produce an effective Ndc80-MT dissociation rate equal to the optimal *k*_d,eff_. Thus, the balance condition can be determined by calculating the effective dissociation rate of Ndc80-MT attachments and setting it equal to the optimal *k*_d,eff_.

**Fig 7:**
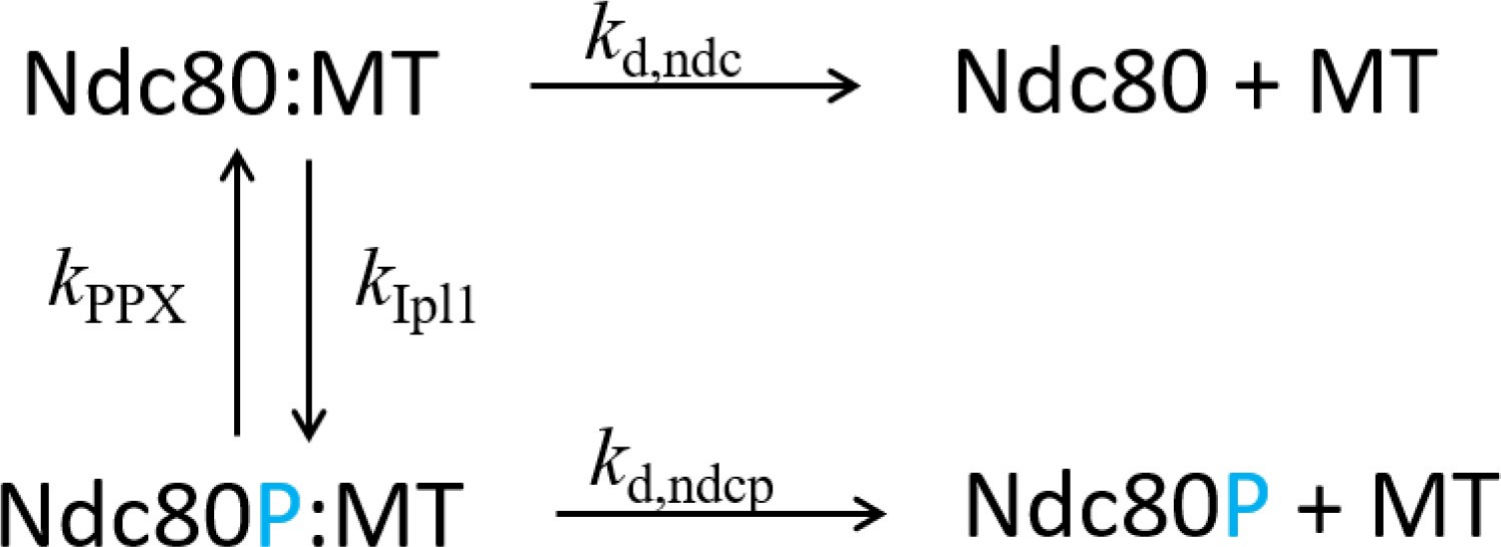
Dissociation scheme of Ndc80-MT attachment.

To calculate the effective dissociation rate of Ndc80-MT attachments (*k*_eff_), we use the scheme shown in Fig 7. The rate constants *k*_d,ndc_ and *k*_d,ndcp_ are the dissociation rates of Ndc80:MT and Ndc80P:MT, respectively

Dissociation of Ndc80 from MT can occur from the states Ndc80:MT or Ndc80P:MT. If we define the rate of dissociation as the inverse of mean first passage time, then the dissociation rate starting from the state Ndc80:MT is given by (see S1 Text in S2 File).

$$k_{1}=\frac{k_{d,\mathrm{ndc}}\cdot k_{d,\mathrm{ndcp}}+k_{d,\mathrm{ndc}}\cdot k_{\mathrm{PPX}}+k_{d,\mathrm{ndcp}}\cdot k_{\mathrm{Ipl}1}}{k_{d,\mathrm{ndcp}}+k_{\mathrm{PPX}}+k_{\mathrm{Ipl}1}}.$$ (1)

In the above expression the number of molecules of Ipl1 and PPX have been absorbed in the corresponding rate constants *k*_Ipl1_ and *k*_PPX_. Similarly, the rate of dissociation starting from Ndc80P:MT state is given by

$$k_{2}=\frac{k_{d,\mathrm{ndc}}\cdot k_{d,\mathrm{ndcp}}+k_{d,\mathrm{ndc}}\cdot k_{\mathrm{PPX}}+k_{d,\mathrm{ndcp}}\cdot k_{\mathrm{Ipl}1}}{k_{d,\mathrm{ndc}}+k_{\mathrm{PPX}}+k_{\mathrm{Ipl}1}}.$$ (2)

Assuming phosphorylated and dephosphorylated states of Ndc80 are in equilibrium, the effective dissociation rate of Ndc80-MT attachment can be written as

$$k_{\mathrm{eff}}=\frac{k_{\mathrm{Ipl}1}}{k_{\mathrm{PPX}}+k_{\mathrm{Ipl}1}}\cdot k_{1}+\frac{k_{\mathrm{PPX}}}{k_{\mathrm{PPX}}+k_{\mathrm{Ipl}1}}\cdot k_{2}.$$ (3)

By setting *k*_eff_ equal to optimal *k*_d,eff_, i.e.,

$$k_{\mathrm{eff}}=\mathrm{optimal}\;k_{d,\mathrm{eff}}=\frac{k_{\mathrm{Ipl}1}}{k_{\mathrm{PPX}}+k_{\mathrm{Ipl}1}}\cdot k_{1}+\frac{k_{\mathrm{PPX}}}{k_{\mathrm{PPX}}+k_{\mathrm{Ipl}1}}\cdot k_{2},$$ (4)

we get a criterion from which the kinase-to-phosphatase balance point (*k*_Ipl1_/*k*_PPX_) can be determined.

**Fig 8:**
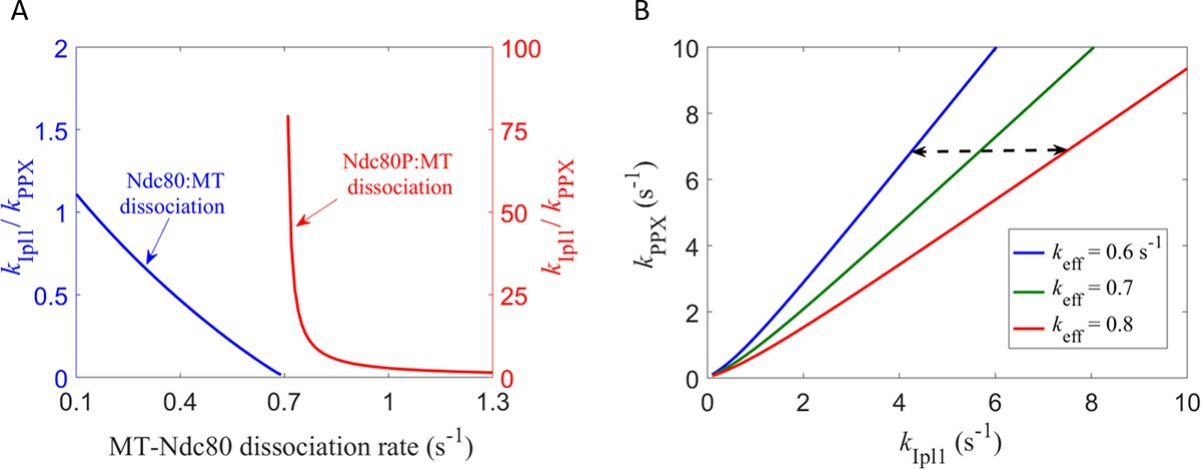
Dependence of kinase-phosphatase balance point on MT-Ndc80 dissociation rates. **(A)** The blue curve corresponds to variation in the Ndc80:MT dissociation rate when the Ndc80P:MT dissociation rate is fixed at 1.5 s^−1^. As the Ndc80:MT dissociation rate is increased towards the optimal value of *k*_d,eff_ (0.7 s^−1^), the balance point drops to zero. The red curve corresponds to variation in the Ndc80P:MT dissociation rate, when the Ndc80:MT dissociation rate is fixed at 0.1 s^−1^. As the Ndc80P:MT dissociation rate is decreased towards optimal *k*_d,eff_, the balance point becomes much larger than one. **(B)** Curves on which the effective MT-Ndc80 dissociation rate *k*_eff_ is constant. The curves were calculated using Eq 3. At a fixed value of *k*_PPX_, the interval between the red and the blue curves is the range of *k*_Ipl1_ over which the biorientation probability is high (see Fig 5). This range increases with increasing *k*_PPX_.

Fig 8A shows the dependence of the kinase-to-phosphatase balance point on the MT-Ndc80 dissociation rates, calculated using Eq 4. When the Ndc80:MT dissociation rate (*k*_d,ndc_) is increased to the optimal *k*_d,eff_ value (0.7 s^−1^ for *x* = 4 case), the balance point drops to zero. On the other hand, when the Ndc80P:MT dissociation rate (*k*_d,ndcp_) is decreased to 0.7 s^−1^, the balance point increases to infinity. These trends can be understood by considering the following limiting cases: (a) as *k*_d,ndc_ → 0.7 s^−1^ and *k*_Ipl1_/*k*_PPX_ → 0, all Ndc80 molecules will be in the unphosphorylated state (see Fig 7), and the effective MT-Ndc80 dissociation rate would approach *k*_d,ndc_ = 0.7 s^−1^ = the optimal *k*_d,eff_ value; conversely, (b) as *k*_d,ndcp_ → 0.7 s^−1^ and *k*_Ipl1_/*k*_PPX_ → ∞,all Ndc80 molecules will be in the phosphorylated state (see Fig 7), and the effective MT-Ndc80 dissociation rate would approach *k*_d,ndcp_ = 0.7 s^−1^ = the optimal *k*_d,eff_ value. This analysis shows that the kinase-to-phosphatase balance point crucially depends on the MT-Ndc80 dissociation rates and the optimal *k*_d,eff_ value.

We also analyzed how the absolute values of kinase and phosphatase activities affect the biorientation process. Fig 8B shows the curves at which *k*_d,eff_ is equal to 0.6, 0.7, 0.8 s^−1^. For these values the biorientation probability for the ‘4 Ndc80’ case is high (Fig 6A). The dashed double arrow shows the range of *k*_Ipl1_ over which biorientation can occur with probability close to one. We make the qualitative observation that, with increasing values of *k*_PPX_, the range of *k*_Ipl1_ over which biorientation can occur with high probability increases.

### Statistics of amphitelic-to-monotelic transitions

The Ipl1-dependent destabilization of MT-Ndc80 attachments is responsible for error correction. The same mechanism is also responsible for transitions from the low-tension amphitelic state to the monotelic state (see Fig 1). These transitions lower the probability of reaching biorientation [40]. Here we calculate the statistics of amphitelic-to-monotelic transitions made by KTs before reaching biorientation and its dependence on the absolute values of kinase and phosphatase activities. The calculation method is described in SI (see S2 Text in S2 File).

The average number of amphitelic-to-monotelic transitions in a single simulation run is shown in Table 2. The values of kinase (*k*_Ipl1_) and phosphatase (*k*_PPX_) activities are chosen so that *k*_eff_, given in Eq 3, takes the value 0.7 s^−1^ (the optimal value of *k*_d,eff_ for *x* = 4 case). As the absolute values of kinase and phosphatase activities are increased, while keeping *k*_eff_ the same, the number of amphitelic-to-monotelic transitions drops. The standard deviations in the number of transitions show that the drop is statistically significant. The number of transitions was converted into probability by using *P* = *N*/(1+*N*), where *N* is the number of amphitelic-to-monotelic transitions and 1 represents the single amphitelic-to-biorientation transition in each run. At higher absolute values of the activities, the probability of amphitelic-to-monotelic transition drops from 0.39 to 0.34. Thus, we find that even when the probability of amphitelic-to-monotelic transition is ~1/3, biorientation can be achieved with high probability (the measured error rate was ~10^−4^ per chromosome).

**Table 2:** Mean number of transitions (*N*) from amphitelic (amp) to monotelic (mon) state at two different kinase-phosphatase balance points*.

*k*_Ipl1_ (s^−1^)	*k*_PPX_ (s^−1^)	*N* (amp → mon)	*P* (amp → mon)	1−*P*_bio_ at 10 min
1	0.9	0.64 (sd = 0.02)	0.39	3.2×10^−4^
5	6	0.51 (sd = 0.02)	0.34	3×10^−4^

*The number of transitions is converted into probability using *P* = *N*/(1+*N*). At higher absolute values of activities, the probability of amphitelic to monotelic transition drops.

The above analysis is related to the ‘initiation problem of biorientation’ which addresses how low-tension amphitelic attachments progress to high-tension amphitelic attachment (biorientation) without going back to the monotelic state. In a previous model [23], with a focus on dynamics of phosphorylation events at the KT, it was found that requiring a large number of phosphorylation events at multiple KT sites can create a delay in KT-MT detachment, and thereby facilitate the biorientation of KTs. In our model, the multiplicity of Ndc80 interactions with a MT enhances the time spent by KTs in the low-tension amphitelic state.

### Effect of kinetochore orientation on biorientation of KTs

Along with the Ipl1-dependent method of error correction, geometrical factors can also contribute to biorientation of KTs. It has been proposed that MT attachment from one spindle pole orients the sister KT in a way that attachment from the opposite spindle pole becomes more probable [41]. Such geometrical orientation of KTs can promote biorientation by reducing the chances of forming syntelic attachments. We asked what are the relative contributions of Ipl1-dependent MT-KT detachment and KT geometrical orientation to achieving biorientation?

**Fig 9:**
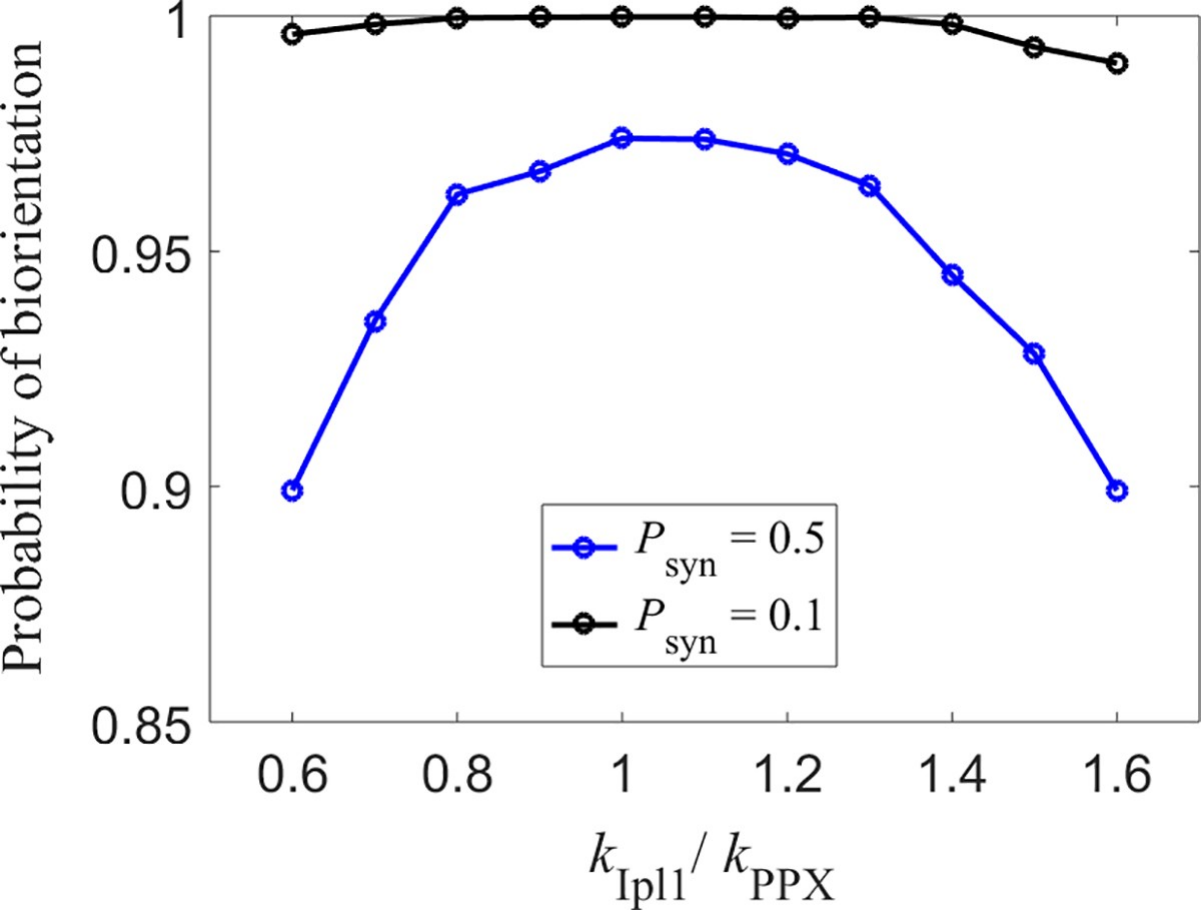
Effect of geometrical orientation of KTs on biorientation probability. Probability of biorientation within 10 min for different values of *P*_syn_. KTs start in unattached state. *P*_syn_ = 0.5 corresponds to the case in which KTs in monotelic state transition to syntelic and amphitelic states with equal probability. The lowest error rate in this case is 4.5×10^−2^ per chromosome (occurs at *k*_Ipl1_/*k*_PPX_ = 1). *P*_syn_ = 0.1 corresponds to the case in which KTs in monotelic state transition to amphitelic state 10 times more often than syntelic state. The error rate in this case at *k*_Ipl1_/*k*_PPX_ = 1 is 10^−4^ per chromosome.

To study the effect of geometrical orientation, we start the KTs in the unattached state and vary the parameter, *P*_syn_, which specifies the ratio of transition between monotelic → syntelic and monotelic → amphitelic states. In Fig 9 we compare the results for *P*_syn_ = 0.1 with results for *P*_syn_ = 0.5, representing the case in which there is no geometrical bias preventing the formation of syntelic attachments, i.e., the transition probabilities from monotelic-to-syntelic and monotelic-to-amphitelic states are equal. For *P*_syn_ = 0.5, the maximum of biorientation probability is approximately 0.9614, which corresponds to an error rate of 1−*P*_bio_ = 4.5×10^−2^. For *P*_syn_ = 0.1, at the same value of *k*_Ipl1_/*k*_PPX_, the error rate is 10^−4^ per chromosome. Thus, we find that, in the absence of geometrical orientation of KTs, the error rate is much larger than the mis-segregation rate observed in budding yeast (between 10^−5^ and 10^−4^). In the presence of geometrical orientation, the error rate drops by a factor of ~500. Thus, we conclude that geometrical orientation plays a significant role in biorientation of KTs.

### Coupling between error correction and SAC

Here we analyze the coupling between error correction and the SAC within the framework of our model. The coupling is quantified by the number of Mps1 molecules bound to the kinetochore, the number of phosphorylated MELT motifs, and the number of phosphorylated Bub molecules bound to the kinetochore (a measure of SAC signal strength). These quantities are defined as the time-average occupancy of the corresponding states, i.e.,

#Mps1 = 〈#Ndc80:Mps1 + #Ndc80P:Mps1〉,
#MELT =〈#MELTP + #MELTP:Bub + #MELTP:BubP〉, and
#BubP = 〈#MELTP:BubP〉.

The method for calculating these quantities is described in SI (see S3 Text in S2 File).

The average number of Mps1 molecules binding per KT in a single simulation run (syntelic to biorientation) was 1.1, which is much smaller than its maximum capacity of 5 (Table 3). The binding of Mps1 was low because of the competition between Mps1 and MT for binding to Ndc80.

**Table 3:** Average number of Mps1 and SAC signaling proteins bound per KT*.

#PP1 per KT	#Mps1 per KT	#MELTP per KT	#BubP per KT
0	1.1	29	5.5
1.1	1.1	19	2.5
5	1.1	17	1.5

^*^The average number of PP1 molecules was chosen to analyze SAC signal strength in the following three cases: PP1 activity much smaller than, equal to, and much larger than Mps1 activity. The calculations were done at the kinase-phosphatase balance point, *k*_Ipl1_ = 1 s^−1^ and *k*_PPX_ = 0.9 s^−1^.

SAC strength is controlled by the opposing activities of Mps1 and PP1. The dynamic regulation of PP1 activity (through binding to the RVSF motif) and its strength compared to Mps1 activity are not well understood. In the absence of this information, we consider cases where the PP1 activity is much less than, equal to, and much greater than the Mps1 activity (Table 3). In the absence of PP1 activity, as expected, the number of phosphorylated MELT motifs per KT was close to the maximum possible value of 30. However, the number of phosphorylated BubP molecules bound to KT (5.5 per KT) was much smaller than the maximum capacity of 30, which is consistent with experimental data [30]. Increasing the forward rate of Bub binding by a factor of five increased the number of Bub molecules bound per KT to 9.5, still significantly below the maximum capacity. For PP1 activity equal to or much greater than Mps1 activity, the average number of BubP molecules bound per KT dropped to 2.5 and 1.5, respectively.

For the above analysis, kinase and phosphatase activities, *k*_Ipl1_and *k*_PPX_, were chosen to be balanced. For kinase activity below the balance point value, the #Mps1 per KT became even smaller. For example, for *k*_Ipl1_ = 0.8 s^−1^ and *k*_PPX_ = 0.9 s^−1^, we found #Mps1 per KT = 0.73, #MELTP per KT = 29, and #BubP per KT = 8.9. On the other hand, for Ipl1 activity above the optimal value (*k*_Ipl1_ = 1.2 s^−1^ and *k*_PPX_ = 0.9 s^−1^), we found #Mps1 per KT = 1.4, #MELTP per KT = 29, and #BubP per KT = 12. As expected, in the latter case the SAC strength is higher.

## Discussion

The fidelity of chromosome segregation is guarded by two coupled mechanisms: error correction in kinetochore-microtubule (KT-MT) attachments and the spindle assembly checkpoint (SAC). The error correction mechanism removes erroneous attachments between KTs and MTs, and the SAC delays the onset of anaphase until all KTs are properly attached to MTs. Since in budding yeast the numbers of molecules involved in these mechanisms are relatively small, systems-level stochastic models are needed to understand the complex dynamics of chromosome biorientation processes. In this paper we present such a model of KT-MT attachment dynamics via the protein Ndc80.

In budding yeast, phosphorylation of Ndc80 by Ipl1 destabilizes kinetochore-microtubule interaction to facilitate the release of erroneous attachments. The role of an opposing phosphatase is to keep the phosphorylation of Ndc80 sub-maximal, so that after error correction is complete, the initial low-tension amphitelic KT-MT attachments can be stable. So far, a quantitative understanding of kinase-phosphatase balance has been missing. Through numerical simulations and derivation of an analytical formula for the balance point, we show that the balance point depends on the MT-Ndc80 dissociation rates and the parameter related to tension-mediated irreversible transition of sister chromatids from low-tension amphitelic states to the high-tension biorientation state. We found that, depending on the values of the parameters, the kinase-phosphatase balance point can be much smaller or larger than 1. Our analysis highlights the complexity of the question and shows that determining the precise value of the balance point is not straightforward. The analytical formula for the balance point was derived assuming Ndc80 molecules can exist in two phosphorylation states. The same analysis can be extended to derive a formula for the kinase-phosphatase balance point when multiple phosphorylation states are present.

During mitosis a pair of sister KTs transition between different attachment states. The number of transitions from a low-tension amphitelic state to the monotelic state is particularly interesting as it depends on how tension is generated between sister KTs and how that tension affects the dynamics of Ndc80-MT attachment. To the best of our knowledge, the statistics of amphitelic-to-monotelic transitions in budding yeast has not yet been measured experimentally. We found that at the kinase-phosphatase balance point, the probability of this transition is approximately 1/3 and the error rate is approximately 10^−4^ per chromosome. This shows that, in budding yeast, biorientation can be achieved even when the amphitelic-to-monotelic transition probability is approximately 1/3 (significantly larger than zero).

When one KT is attached to a spindle pole, the sister KT has a high probability to face the opposite spindle pole. We calculated the probability of biorientation when that geometrical constraint is relaxed, and found the error rate to be 4.5×10^−2^ per chromosome; a rate that is much higher than the experimentally observed chromosome mis-segregation rate of 10^−5^ to 10^−4^ per chromosome. When the geometrical constraint was introduced, the error rate dropped 500-fold to approximately 10^−4^ per chromosome. This suggests that, in budding yeast, the geometrical orientation of chromosomes plays a significant role in biorientation of chromosomes.

The model presented in this manuscript is a first attempt towards developing a realistic, systems-level, stochastic model of the dynamics of error correction in KT-MT attachments. Our model, though simple, can be used to calculate statistical properties of the KT-MT attachment process, including the distribution of biorientation times, the probability of transition between specific attachment states, and the KT-occupancy of SAC proteins like Mps1 and Bub1. Currently, the precise values of parameters in the model (e.g., the numbers of molecules of different proteins, the binding/unbinding rates of proteins, and phosphorylation/dephosphorylation rates) are not available from experiments. Nevertheless, we think, our model provides a basic framework for future development of more detailed and accurate, system-level models of error correction and its coupling to the spindle assembly checkpoint.

## Supporting information

S1 File (TXT)

S2 File (DOCX)
