# Supplementary material for: A stochastic model for error correction of kinetochore-microtubule attachments in budding yeast
Source: PLoS One. 2020 Aug 6;15(8):e0236293. doi: 10.1371/journal.pone.0236293 (PMC7410253; doi:10.1371/journal.pone.0236293)
Supplement: S2 File — (DOCX) [file pone.0236293.s002.docx]

**A stochastic model for error correction of kinetochore-microtubule attachments in budding yeast**

by

Anand Banerjee, Neil Adames, Jean Peccoud and John J. Tyson

| Table S1 | List of species in the model and their initial values used in simulations | Page 2 |
| --- | --- | --- |
| Table S2 | List of parameter values used in simulations | Page 4 |
| Table S3 | Percentage of time spent by KTs in different KT-MT attachment states in the simplified model | Page 6 |
| Table S4 | Probability of sister KTs reaching biorientation within 10 min, starting from the syntelic attachment state. | Page 7 |
| Figure S1 | Mean biorientation time as a function of the effective dissociation constant *k*_d,eff_. | Page 8 |
| Text S1 | Calculation of effective dissociation rate of Ndc80-MT attachment | Page 9 |
| Text S2 | Calculation of average number of transitions of sister KTs between different attachment states | Page 12 |
| Figure S3 | Time evolution of attachment status of sister KTs at two balance points of kinase-phosphatase activities | Page 13 |
| Text S3 | Calculation of #BubP and #Mps1 | Page 14 |
|  | References | Page 15 |

**Table S1: List of species in the model and their initial values used in simulations.** The same initial conditions were used for both of the sister KTs.

| **Species** | **Description** | **Initial value (# molec)** |
| --- | --- | --- |
| MELT | MELT motif on Spc105 | 10 |
| MELTP | Phosphorylated MELT motif | 10 |
| MELTP:Bub | Phosphorylated MELT motif bound to Bub | 10 |
| Ndc1 | Unbound Ndc80 | 0 |
| Ndc1p | Phosphorylated unbound Ndc80 | 0 |
| Ndc1:Mps1 | Ndc80 bound to Mps1 | 0 |
| Ndc1P:Mps1 | Ndc80 phosphorylated and bound to Mps1 | 0 |
| MT:Ndc1 | MT bound to one Ndc80 | 0 |
| MT:Ndc2 | MT bound to 2 Ndc80s | 0 |
| MT:Ndc3 | MT bound to 3 Ndc80s | 0 |
| MT:Ndc4 | MT bound to 4 Ndc80s | 0 |
| MT:Ndc5 | MT bound to 5 Ndc80s | 1 |
| MT:NdcP | MT bound to 1 Ndc80p | 0 |
| MT:Ndc2P | MT bound to 2 Ndc80p | 0 |
| MT:Ndc3P | MT bound to 3 Ndc80p | 0 |
| MT:Ndc4P | MT bound to 4 Ndc80p | 0 |
| MT:Ndc5P | MT bound to 5 Ndc80p | 0 |
| MT:Ndc1:Ndc1P | MT bound to 1 Ndc80 and 1 Ndc80p | 0 |
| MT:Ndc2:NdcP | MT bound to 2 Ndc80 and 1 Ndc80p | 0 |
| MT:Ndc1:Ndc2P | MT bound to 1 Ndc80 and 2 Ndc80p | 0 |
| MT:Ndc1:Ndc3P | MT bound to 1 Ndc80 and 3 Ndc80p | 0 |
| MT:Ndc2:Ndc2P | MT bound to 2 Ndc80 and 2 Ndc80p | 0 |
| MT:Ndc3:Ndc1P | MT bound to 3 Ndc80 and 1 Ndc80p | 0 |
| MT:Ndc4:Ndc1P | MT bound to 4 Ndc80 and 1 Ndc80p | 0 |
| MT:Ndc3:Ndc2P | MT bound to 3 Ndc80 and 2 Ndc80p | 0 |
| MT:Ndc2:Ndc3P | MT bound to 2 Ndc80 and 3 Ndc80p | 0 |
| MT:Ndc1:Ndc4P | MT bound to 1 Ndc80 and 4 Ndc80p | 0 |

**Table S2: List of parameter values used in simulations.**

| **Parameter** | **Description** | **Value** |
| --- | --- | --- |
| Mps1_T_ | Number of molecules of Mps1 in nucleus | 100 |
| Bub_T_ | Number of molecules of Bub in nucleus | 100 |
| #Spc105 | Number of Spc105 on each kinetochore | 5 |
| #MELT | Number of MELT repeats on each Spc105 | 6 |
| #Ndc80 | Number of Ndc80 on each kinetochore | 5 |
| *k*_Mps1_ | Phosphorylation rate of MELT by Mps1 | 1 s^−1^ |
| *k*_fbub_ | On rate for Bub binding to MELT motif | 0.001 s^−1^ |
| *k*_dbub_ | Dissociation rate of Bub from MELT motif | 0.1 s^−1^ |
| *k*_fmps1_ | On rate of Mps1 binding to Ndc80 | 0.001 s^−1^ |
| *k*_dmps1_ | Dissociation rate of Mps1 from Ndc80 | 0.07 s^−1^ |
| *k*_fndc_ | On rate of Ndc80 binding to MT | 2 s^−1^ |
| *k*_MT_ | Rate of attachment of new microtubule | 0.01 s^−1^ |

The numbers of molecules of Mps1 and Bub are chosen to be 100, which, in a nuclear volume of approximately 4 μm^3^, is equivalent to a concentration of 40 nM. The phosphorylation and dephosphorylation rates of kinases and phosphatases are not well known. We choose all of them to be 1 s^−1^. We estimate the forward binding rate of Mps1 to Ndc80 and of Bub to MELT from their diffusion-limited rate of binding to the KT. Assuming the KT to be a disc of radius $r={10}^{-2}\text{μm}$, the diffusion-limited rate is given by

$$\frac{4Dr}{V}=\frac{4\cdot1({\mu m}^{2}/s)\cdot{10}^{-2}\mu m}{4{\mu m}^{3}}= {10}^{-2}s^{-1},$$

where *D* ≈ 1 μm^2^/s is the diffusion constant of a protein inside the nucleus, and *V* = 4 μm^3^ is the volume of the nucleus. Since the binding sites of these proteins occupy only a small part of the KT, we choose a value of ${10}^{-3}s^{-1}$ for *k*_fbub_ and *k*_fmps1_, which is 10 times smaller than the above estimate. The dissociation constant of Bub bound to the MELT motif was found to be 70nM (1). Using this value, the dissociation rate of Bub (*k*_dbub_) is calculated to be

$$k_{\mathrm{dbub}}=K_{\mathrm{dbub}}\cdot k_{\mathrm{fbub}}\cdot V\approx0.13 \text{s}^{-1}$$

We choose *k*_dbub_ = 0.1 s^−1^. The turnover rate (half-life) of Mps1 at the kinetochore is known to be 10 s (2); hence, the dissociation rate of Mps1 is estimated to be *k*_dmps1_ = ln(2)/10 = 0.07 s^−1^. The forward rate for Ndc80 binding to microtubule, *k*_fndc_ = 2 s^−1^, was taken from Ref. (3). The attachment rate of new microtubules to KTs in fission yeast is 0.06 s^−1^ (4); here we chose *k*_MT_ = 0.01 s^−1^.

**Table S3: Percentage of time spent by KTs in different KT-MT attachment states in the simplified model.**

| *k*_d,eff_ (s^−1^) | syntelic | unattached | monotelic | amphitelic |
| --- | --- | --- | --- | --- |
| 0.5 | 76 | 0.4 | 17.4 | 6.2 |
| 0.7 | 47 | 3 | 30 | 20 |
| 0.9 | 18 | 11 | 43 | 28 |

The analysis was done for the ‘4 Ndc80’ case. The optimal value of *k*_d,eff_ (at which the probability of biorientation is highest) is 0.7 s^−1^ in this case. We find that below the optimal value the KTs spend the largest fraction of time (76%) in the syntelic state; whereas, above the optimal value the largest amount of time is spent in the monotelic state (43%). Based on this observation we conclude that, below the optimal value, the biorientation probability drops because correction of syntelic attachments becomes inefficient, and above the optimal value, the probability drops because correct attachments are destabilized too often.

**Table S4. Probability of sister KTs reaching biorientation within 10 min, starting from the syntelic attachment state.**

| *k*_d,ndc_ (s^−1^) | *k*_d,ndcp_ (s^−1^) | *k*_Ipl1_ (s^−1^) | *k*_PPX_ (s^−1^) | 1-*P*_bio_ |
| --- | --- | --- | --- | --- |
| 0.7 | 0.7 | 0 | 0 | 4×10^−4^ |
| 0.5 | 0.6 | 1 | 0 | 4×10^−3^ |
| 0.8 | 0.9 | 0 | 1 | 5×10^−3^ |

Ndc80:MT and Ndc80P:MT dissociation rates (*k*_d,ndc_ and *k*_d,ndcp_, respectively) are chosen to be either smaller or larger than the optimal value (0.7 s^−1^ for *x* = 4 case) The *k*_Ipl1_ and *k*_PPX_ values are the kinase and phosphatase activities at which the highest biorientation probability was observed. The last column shows the error rate (1-*P*_bio_). When both dissociation rates are smaller or larger than the optimal dissociation rate (0.7 s^−1^), the error rate increases significantly. This suggests that *k*_d,ndc_ should be smaller and *k*_d,ndcp_ should be greater than the optimal value of *k*_d,eff_.

**
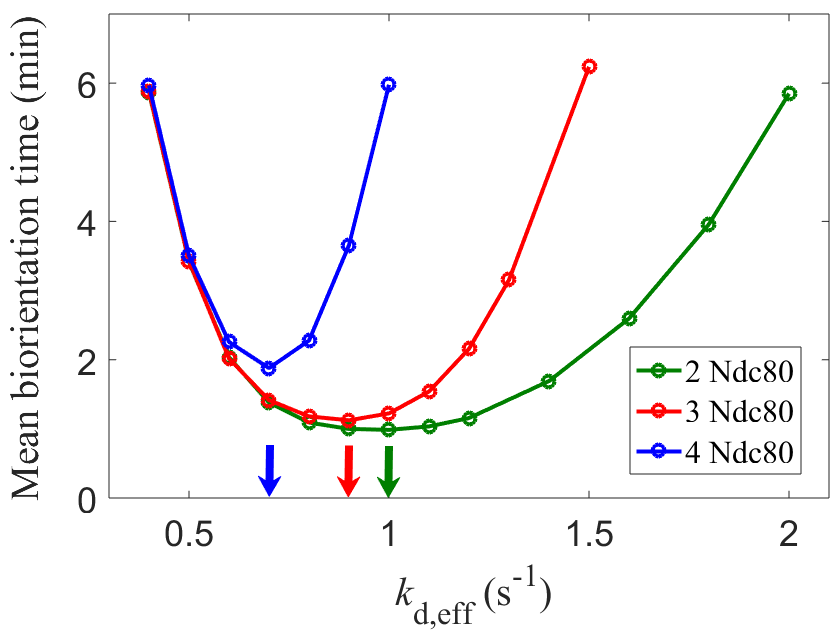
**

**Figure S1. Mean biorientation time as a function of the effective dissociation rate *k*_d,eff_.** Different curves correspond to different numbers of Ndc80s required for biorientation. As this number is lowered, the average biorientation time drops. The minimum in biorientation time is used to identify the optimal values of *k*_d,eff_ (shown with arrows).

**Text S1: Calculation of effective dissociation rate of Ndc80-MT attachment**

Let *x* and *y* denote the probabilities that the system is in states Ndc80:MT and Ndc80P:MT, respectively. Based on the scheme below, the rate of change of probabilities can be written as


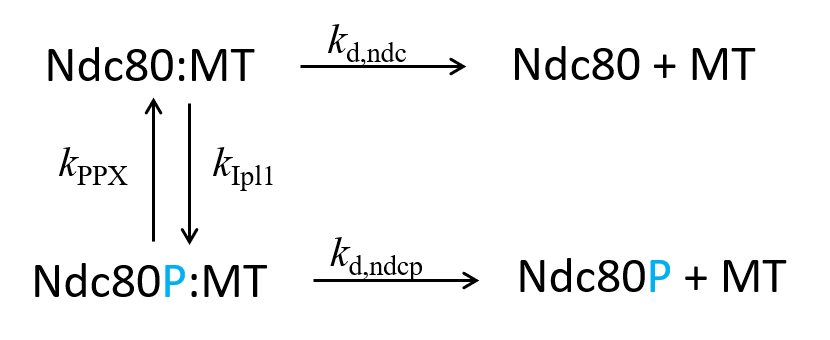

$$\dot{x}\left( t \right)= -\left( k_{d,ndc}+k_{Ipl1}\cdot\#Ipl1 \right)\cdot x +k_{\mathrm{PPX}}\cdot\#PPX\cdot y= -\alpha\cdot x + k_{\mathrm{PPX}}\cdot\#PPX\cdot y$$

$$\dot{y}\left( t \right)= k_{Ipl1}\cdot\#Ipl1\cdot x - \left( k_{d,ndcp}+k_{\mathrm{PPX}}\cdot\#PPX \right)\cdot y= k_{Ipl1}\cdot\#Ipl1\cdot x - \beta\cdot y$$

where

$\alpha= k_{d,ndc}+k_{Ipl1}\cdot\#Ipl1$ and $\beta= k_{d,ndcp}+k_{\mathrm{PPX}}\cdot\#PPX$.

Suppose the system starts in the Ndc80:MT state, i.e., $x\left( 0 \right)=1, y\left( 0 \right)=0.$ Taking Laplace transform of the equations and setting $\#Ipl1=\#PPX=1$, we get

$$s\cdot\tilde{x}\left( s \right)-1= -\alpha\cdot\tilde{x} + k_{\mathrm{PPX}}\cdot\tilde{y}$$

$$s\cdot\tilde{y}\left( s \right)=k_{Ipl1}\cdot\tilde{x} - \beta\cdot\tilde{y}$$

Solving for the Laplace transforms we get

$$\tilde{x}\left( s \right)= \frac{s+\beta}{\left( s+\alpha\right)\cdot\left( s+\beta\right)-\gamma} , \tilde{y}(s)= \frac{k_{Ipl1}}{\left( s+\alpha\right)\cdot\left( s+\beta\right)-\gamma}$$

The probability density of exit times and its Laplace transform can be written as

$$\varphi\left( t \right)=k_{d,ndc}\cdot x\left( t \right)+k_{d,ndcp}\cdot y\left( t \right) \mathrm{and} \tilde{\varphi}\left( s \right)= k_{d,ndc}\cdot\tilde{x}\left( s \right)+k_{d,ndcp}\cdot\tilde{y}(s)$$

Substituting for the Laplace transforms we get

$$\tilde{\varphi}\left( s \right)= \frac{k_{d,ndc}\cdot\left( s+\beta\right)+k_{d,ndcp}\cdot k_{Ipl1}}{\left( s+\alpha\right)\cdot\left( s+\beta\right)-\gamma}$$

The mean first-passage time is given by

$$\tau_{1}= -\frac{\partial\tilde{\varphi}\left( s=0 \right)}{\partial s}= \frac{k_{d,ndcp}+k_{\mathrm{PPX}}+k_{Ipl1}}{k_{d,ndc}\cdot k_{d,ndc1}+k_{d,ndc}\cdot k_{\mathrm{PPX}}+k_{d,ndcp}\cdot k_{Ipl1}}$$

The inverse of $\tau$ is taken as the effective dissociation rate. The same analysis with initial conditions, $x\left( 0 \right)=0, y\left( 0 \right)=1$, i.e., the systems starts in the Ndc80P:MT state, yields

$$\tilde{x}\left( s \right)= \frac{k_{\mathrm{PPX}}}{\left( s+\alpha\right)\cdot\left( s+\beta\right)-\gamma} , \tilde{y}(s)= \frac{s+\alpha}{\left( s+\alpha\right)\cdot\left( s+\beta\right)-\gamma}$$

$$\tilde{\varphi}\left( s \right)= \frac{k_{d,ndc}\cdot k_{\mathrm{PPX}}+k_{d,ndcp}\cdot\left( s+\alpha\right)}{\left( s+\alpha\right)\cdot\left( s+\beta\right)-\gamma},$$

and

$$\tau_{2}= \frac{k_{d,ndc}+k_{\mathrm{PPX}}+k_{Ipl1}}{k_{d,ndc}\cdot k_{d,ndcp}+k_{d,ndc}\cdot k_{\mathrm{PPX}}+k_{d,ndcp}\cdot k_{Ipl1}}$$

**Text S2: Calculation of average number of transitions of sister KTs between different attachment states**

The traces shown in Fig. S3 were used to calculate the statistics of attachment. The fraction of time in each state, for one trace, was calculated by summing up the time in each state and dividing it by the length of the trace (time to reach biorientation). The individual fractions were then averaged over 10^4^ realizations. For example, the fraction of time in the syntelic state was calculated as

$$f_{\mathrm{syn}}=\frac{1}{10000}\cdot\sum_{i=1}^{10000} f_{i}$$

where $f_{i}$ is the fraction of time in syntelic state in the *i*^th^ trace. The average number of transitions between different states was determined by counting the number of transitions between different states in 10^4^ realizations and dividing that number by 10^4^. For example, the average number monotelic to syntelic transitions were calculated as

$$n_{\mathrm{mon}\to\mathrm{syn}}=\frac{1}{10000}\cdot\sum_{i=1}^{10000} n_{i}$$

where *n_i_* is the number of mon $\to$ syn transitions in the *i*^th^ trace.

**
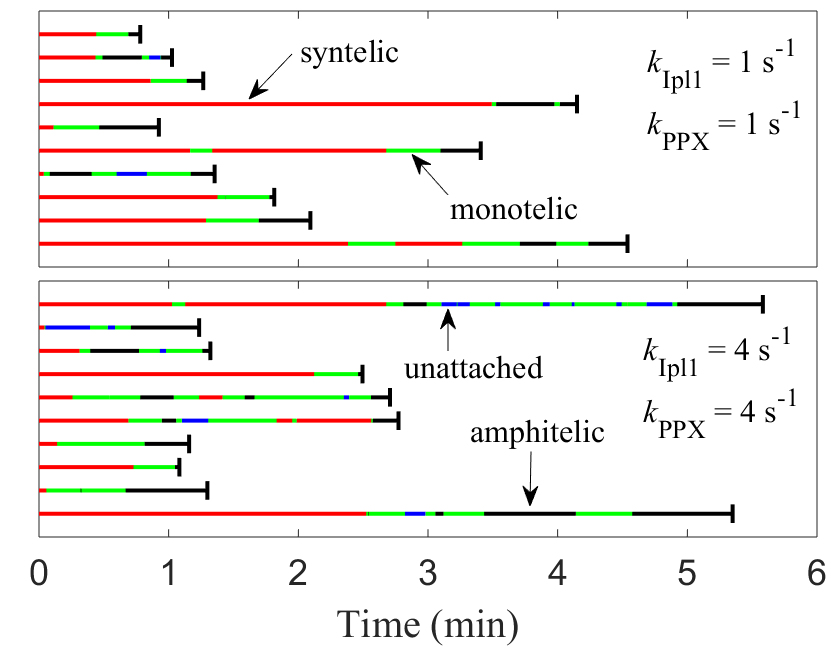
**

**Figure S2. Time evolution of the attachment status of sister KTs at two balance point of kinase-phosphatase activities.** Different traces correspond to different realizations. The attachment status is shown with different colors: syntelic (red), monotelic (green), unattached (blue), and amphitelic (black). The starting point of each trace is syntelic state and the end point is the biorientation state.

**Text S3: Calculation of #BubP and #Mps1**

#BubP and #Mps1 are the average occupancy of the state producing the SAC signal and Mps1 molecules bound to Ndc80, respectively. They are defined as

#BubP = 〈#MELTP:BubP〉

#Mps1 = 〈#Ndc80:Mps1 + #Ndc80P:Mps1〉 ,

where the # symbol indicates number of molecules. For a single trace, as shown in Fig S3, we calculated the average as

$$<X> = \frac{1}{T}\cdot\sum_{i} X_{i}*{\Delta t}_{i},$$

where $X$ = #MELTP:BubP or #Ndc80:Mps1 + #Ndc80P:Mps1, and $X_{i}$ is the occupancy number of state $X$ between the time interval ${\Delta t}_{i}=t_{i}- t_{i-1}$. The time $T$ corresponds to the time needed to reach biorientation or 10 min. Finally, the single-trace-average was averaged over 10^3^ traces to determine the final number.

**References**

1. Aravamudhan P, Chen R, Roy B, Sim J, Joglekar AP. Dual mechanisms regulate the recruitment of spindle assembly checkpoint proteins to the budding yeast kinetochore. *Mol Biol Cell*. 2016; 27(22):3405-17.

2. Howell BJ, Moree B, Farrar EM, Stewart S, Fang G, Salmon ED. Spindle checkpoint protein dynamics at kinetochores in living cells. *Curr Biol*. 2004; 14(11):953-64.

3. Zaytsev AV, Mick JE, Maslennikov E, Nikashin B, DeLuca JG, Grishchuk EL. Multisite phosphorylation of the NDC80 complex gradually tunes its microtubule-binding affinity. *Mol Biol Cell*. 2015; 26(10):1829-44.

4. Gay G, Courtheoux T, Reyes C, Tournier S, Gachet Y. A stochastic model of kinetochore-microtubule attachment accurately describes fission yeast chromosome segregation. *J Cell Biol*. 2012; 196(6):757-74.
